# Periodontal Health and Caries Prevalence Evaluation in Patients Affected by Parkinson's Disease

**DOI:** 10.1155/2012/541908

**Published:** 2012-12-18

**Authors:** Marco Cicciù, Giacomo Risitano, Giuseppe Lo Giudice, Ennio Bramanti

**Affiliations:** ^1^Department of Human Pathology, University of Messina School of Dentistry, 98100 Messina, Italy; ^2^Department of Mechanical Engineering, Facilities, and Infrastructure, Guglielmo Marconi University, Rome, Italy; ^3^Odontostomatology Department, School of Dentistry, University of Messina, 98100 Messina, Italy

## Abstract

Parkinson's disease (PD) is a progressive neurodegenerative disorder related to the loss or absence of dopaminergic neurons in the brain. These deficits result in slowness of movement, tremor, rigidity, and dysfunction of behaviour. These symptoms negatively influence the patient's capability to carry out the daily oral hygiene manoeuvres. The aim of this work is to record the oral health condition of PD patients evaluated at the IRCSS Bonino-Puleio in Messina. The oral health of 45 consecutive PD patients (study group) with neurologic diagnosis based on United Kingdom Brain Bank Criteria has been compared with that of another 45 no PD patients of the same age (control group). The evaluation of the general oral condition was recorded underlining tooth loss, active periodontal disease, and presence of untreated caries. The frequency of untreated caries, periodontal diseases, and missing teeth of the study group was significantly higher than in control group. Based on the data results, clinicians should direct high attention to the oral hygiene of patients with PD, above all at the early stages of the caries or periodontal disease, in order to prevent serious evolution of those pathologic dental conditions that may finally result in the tooth extraction event.

## 1. Introduction 

Parkinson's disease (PD) is a progressive neurodegenerative disorder typically characterized by motor symptoms such as bradykinesia, rigidity, and postural instability with resting tremor. In addition, cognitive impairments are present, even in early disease stages, and predominantly affect executive functions such as planning abilities. Moreover, regarding a high risk for clinical dementia and clinical depression nondemented and nondepressed PD patients manifest themselves subtle cognitive problems, even in the earliest disease stages, which reflect incident pathological dementia and quality of life [1–6]. 

The motility impairment in PD patients is related with the damage and loss of about 60 to 70% of the neurons that memory and release dopamine in the substantia nigra. This disability results on the deletion of the neurotransmitter in the striatum area of the basal ganglia, basic to produce smooth and coordinated body movement [7–9]. Therefore, a large spectrum of no motor signs like hypotension, cardiac dysrhythmias, sweating bladder constipation, and sex dysfunction is typical of the PD Patients [10].

Parkinson tremors usually begin in a hand and then inducing alteration of the movements. Through the disease development, tremor involves the legs, face, tongue, and mandible [2, 11, 12]. 

In patients with Parkinson's disease (PD), chronic levodopa treatment may be related with several dyskinetic movements (levodopa induced dyskinesias (LID)), which are classified according to the type of movement and also in relation with the effect of levodopa.

The association between levodopa and the induction of dyskinesias was recognized soon after the introduction of levodopa [13, 14]. In the past, levodopa therapy was associated with the development of motor complications in about 80% of patients within 5 years of treatment [15, 16]. In patients with young onset PD, the incidence of LID was higher and ensued more rapidly [14, 17]. Currently, with the introduction and widespread use of dopaminergic agonists, the overall treatment exposure to levodopa is decreasing, especially in the first years of treatment; nevertheless, progression of the nigrostriatal deficit will facilitate the onset of LID at a later point in time. Thus, LID continues to be a common and important cause of disability in PD and one of the main reasons for recommending surgical treatment.

The diagnosis of PD is based on patient's anamnesis and medical history. However, the clinical evaluation of the signs by physical examination and in some cases, a positive sustained response to dopaminergic medications are parameters that clinicians should be considered for performing a correct diagnosis. Therefore, instrumental investigation like laboratory tests and imaging studies are not routinely used [6, 8, 18].

Parkinson patient's disability can be classified accordingly to neurological graveness of the diseases. Hoehn and Yahr firstly proposed a scale based on the type of motor symptoms, assist clinicians in staging the disorder [19, 20]. Due to the tremors, the PD patients may reveal difficulty for performing oral hygiene daily maneuvers. Numerous co-factors like motor impairment, dysphagia, apathy, depression, dementia, hypersialorrhea, xerostomia can involve to this disability. In addition, patients also have a progressive relative inability to start voluntary and involuntary movements [6, 8, 11]. The facial expression initiates to be reduced, blinking and swallowing may be associated too. Other clear signs are the incapability of getting dressed, bathing, arising from a sitting position, and the overall sense of weakness. Body muscles motility is not usual and uncontrollable related to an increased muscular tone developed. Autonomic dysfunction of PD patients manifests itself as variations in blood pressure causing cardiac dysrhythmias, excessive sweating, and consequently sexual dysfunction. Insomnia, sleep apnoea, and sleep fragmentation with resultant daytime drowsiness are disability conditions of PD patients [19–21].

Poor dental and periodontal health, conversely, may be a risk factor for progression of associated disease like diabetes mellitus, pulmonary disease, atherosclerosis, cardiovascular disease, and stroke [21–24].

The damaging effects related with this progressive disease can significantly influence life and especially the oral health of those patients. Oral hygiene of PD patients can often be neglected as a result of the disease's evolution. For this reason, the clinicians through the complete knowledge of the disease should perform accurate dental control manoeuvres. Moreover, the general dentists should perform an overview of the patient's PD clinical features, and pharmacological drugs administration in order to increase the patients' PD oral health. The purpose of this investigation is to evaluate the frequencies of periodontal disease and caries, tooth loss in PD patients in relation to oral hygiene condition guiding early intervention and developing new therapies necessary to give PD patients high life quality.

## 2. Patients and Methods 

Between May 2012 and August 2012 the oral condition of periodontal health and caries prevalence were recorded in 45 consecutive 65 to 78 years old patients affected by Parkinson's disease of a mild type in stages 1-2 of Hoehn and Yahr scale [20] (study group). PD Patients were consecutively recruited from IRCSS Neurolesi Bonino-Puleio.

One other 45 patient's group of the same ages was recorded (control). Those consecutive patients were recruited at the Odontostomatolgy Department of Messina Policlinic, where they came for routine dental visit. 

The institutional ethical committee board of IRCCS Centro Neurolesi “Bonino-Pulejo” Messina approved protocol, and all subjects and their proxies provided written informed consent. 

One of the investigators clinically inspected the oral cavity by dental probe using for periodontal deep socket investigation. Clinical investigation was completed by mouth mirror investigation for evaluating the presence of dental caries and the number of missed teeth was recorded for each patient. The teeth involved in previous conservative, endodontic, or prosthetic treatment were not included in the one caries affected. For further investigation all patients underwent X-ray ortopanoramic investigation in order to record also the presence of interproximal caries. All the periodontal pockets deeper than 4 mm were considered pathologic accordingly with the International Periodontal Index [25].

Patients with at least three pathological pockets on two different teeth were classified to have periodontal disease. Clinical pictures of the patients were recorded and included on the study for comparing the two groups. The study PD patients' cognitive functions were assessed by the mini-mental state examination (MMSE) and the motor impairment severity was assessed by the Persson et al. stage [24]. The decayed, missing and filled test (DMFT) was done for all the patients in both groups.

## 3. Results

A total of 90 patients have been examined. The study group involved patients affected by Parkinson's disease (28 women, 17 men) and the control group (10 women, 35 men) recorded no PD patients. The mean ages of PD patients were comparable and similar to the control group. 

### 3.1. Number of Missed Tooth

(The complete dentature accordingly to the International Consensus about the presence of 28 teeth in upper and lower jaw avoiding the presence of the wisdom teeth) [25].

- The number of missed teeth per patient ranged from 10 to 22 in the PD group with a media of 13 missed teeth for person (a total of 330 teeth has been clinically recorded)
- The number of missed teeth per patient ranged from 8 to 23 in the control group with a media of 9 missed teeth for person (a total of 418 teeth has been clinically recorded)

### 3.2. Untreated Caries

- The number of untreated caries per patient ranged from 3 to 18 in the PD group (lesions were recorded on 190 of 330 teeth)
- The number of untreated caries per patient ranged from 6 to 14 in the control group (lesions were recorded on 203 of 418 teeth).

### 3.3. Incidence of Periodontal Disease

Baseline periodontal scores. PPD: probing pocket depth, SBI: sulcus bleeding index, Mob: tooth mobility (accordingly with Loe and Sillness Gingival index (1963) Muhlemann and Son's Sulcus; Bleeding index; and Plaque index) [26, 27].

**Figure 1:**
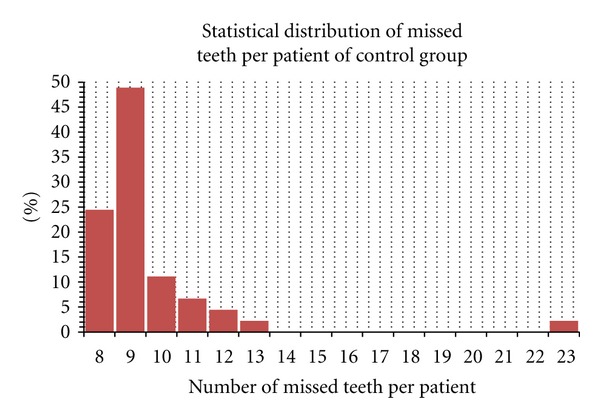
Student's *t*-test performed for number of missed teeth on control Parkinson's patient group.

**Figure 2:**
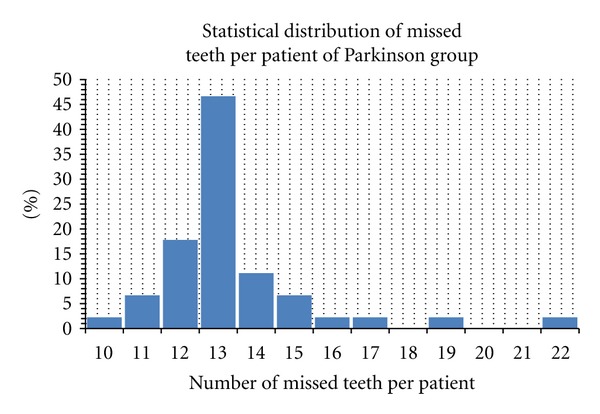
Student's *t*-test performed for number of missed teeth on control group patient.

**Figure 3:**
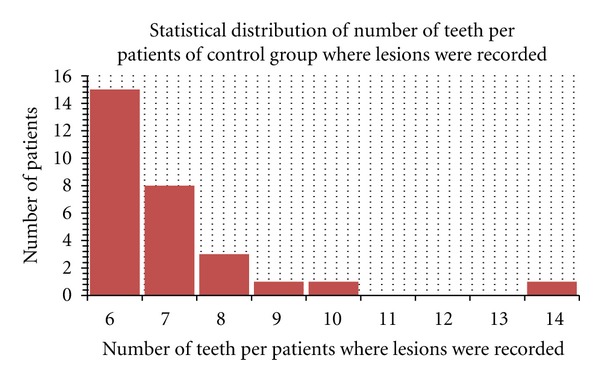
Number of caries recorded in control group.

**Figure 4:**
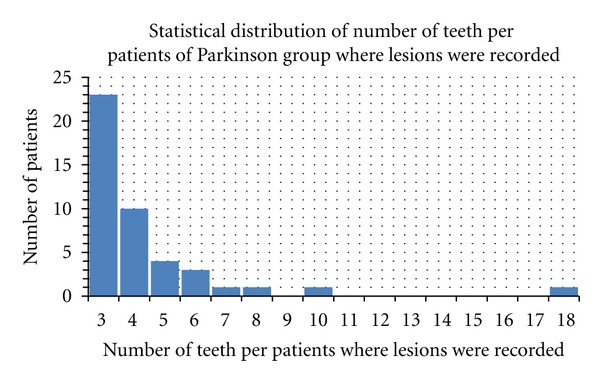
Number of caries recorded in PD group.

**Figure 5:**
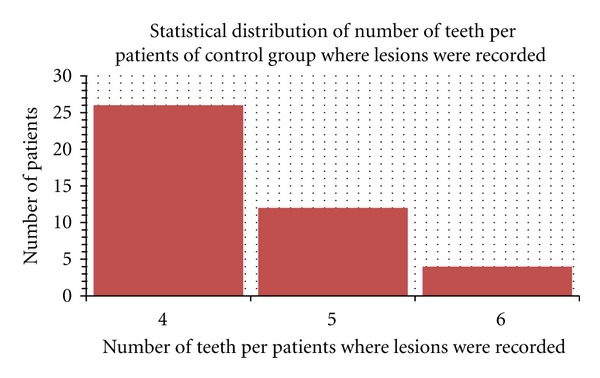
Deep of the sockets recorded in control group.

**Figure 6:**
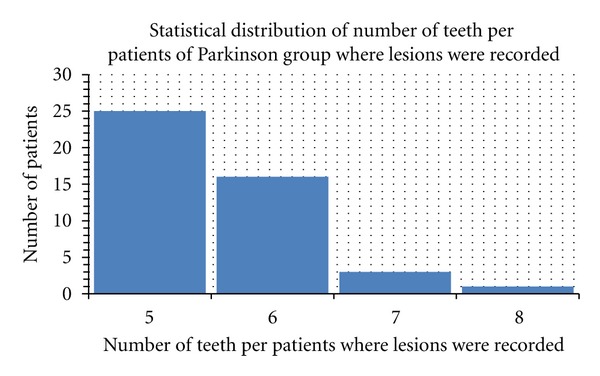
Deep of the sockets recorded in PD group.

**Figure 7:**
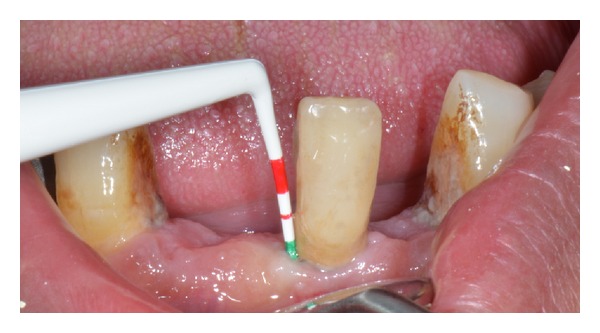
Clinical investigation is performed by periodontal probe in order to evaluate the socket deep.

- A total of the 250 on 330 teeth investigated in the PD study group revealed periodontal pockets ranged from 5 to 8 with a bleeding score positive on pocket probing (92 of 250 teeth with periodontal disease). Severe tooth mobility (Miller class II-III) 23 was recorded on 74 of 250 teeth with periodontal disease.
- A total of 188 of 418 teeth recorded in the control group revealed periodontal pockets ranged from 4 to 6 with negative bleeding score. No severe tooth mobility (Miller class II-III) 23 was recorded in the Control group. (Figures 1, 2, 3, 4, 5, 6 and 7).

## 4. Discussion

Data results clearly showed how the frequencies of missed teeth and periodontal disease were significantly higher in the PD patients group while there was no significant difference between the percentages of untreated caries teeth on both group. Therefore, specifically about the periodontal disease, no marked bleeding and no gingival inflammation signs were observed in the control group. The results of the present study may be principally connected to the clinical patients' oral conditions of the two investigated groups. The similar condition of untreated caries and the same frequency of this pathology in both groups can be related to the sugar diet that older people prefer to assume [28, 29].

Nutrition is an important determinant of health in elderly patients. Over the past decade, the importance of nutritional condition has been increasingly evaluated in numerous morbid conditions including cancer, heart disease, and dementia in persons over the age of sixty-fives; therefore, frequent meals and snacks are suggested by physicians and dieticians. In many cases, the snacks are high in sugar, soft and sticky, favouring plaque formation. Sugar diet will also significantly increase the risk of caries and periodontal disease [29–32]. Moreover, vitamin drinks as substitute of snacks may help maintain proper nutrition and eliminate the caries and periodontal disease predisposing factors.

This study highlights that the maintenance of high oral hygiene is fundamental for patients affected by neurological disease like PD. Results demonstrated that periodontal disease is frequent in patients with PD and regarding the difficulty in plaque removing, it is related to motor and cognitive impairment. Merchant et al. pointed out that the increasing of physical activity and diminished the risk of periodontal disease [33].

The altered ability to have regular oral self-care is connected with impaired manual dexterity, as well as cognitive problems, apathy, depression, and changed motor behaviour and fluctuations. Absent-mindedness associated with dementia may also influence negatively oral hygiene habitude in many individuals with PD [4, 6, 33].

Clinicians should give to the patients with no cognitive impairments instructions in proper tooth brushing and flossing methods that maximize removal of dental plaque. 

The opportunity of this study involving PD patients underlined how there are behavioural changes that may negatively impact dental care. Apathy, depression, and forgetfulness are factors that may influence patients' ability to notice dental problems [12, 22, 24, 32].

Moreover, PD patients seemed to have a decreased appetite. This status, associated with the poor dental hygiene, often increase the tendency to not assume nutrient chewing rich foods like vegetables or meat [31–33]. 

People with Parkinson's may be strongly influenced by dentist prescriptions and suggestions. This group of patients reflects a large load for the health care system because of its occurrence among the increasing proportion of elderly people in Italy. The results of the investigation clearly underlined how the PD patients' age is increasing. For this reason, dentistry and medicine have much to offer patients with this disease, in order to prevent and control caries or periodontal diseases. Topical fluoride gel treatments are commonly used for the plaque control and a daily application may be useful for having better home hygiene in the between of dental visits. Patients of the study seem to accept this kind of treatment cause of easy application and pleasant on tasting. However the interdisciplinary treatment represents the better choice. Moreover to have safe therapeutic strategies, the dentist should consult with the patient's physician to identify any need for modifications of typical treatment practices [34, 35]. 

After recording oral health status in both groups, patient's requests and problems about dental hygiene were underlined and recorded. All the patients were uncomfortable with the mouthwashes, maybe for the fear of choking. Our goal was to have the patient PD total trust and confidence in the dental visits and in the prescription. 

Accordingly to the literature and with our experience, there are numerous procedures in which people with Parkinson's can increase the value of their visits to the dentist, beginning with timing them strategically [29, 33–35]. All the dental visits recorded in the study were performed in the early morning, when the attention grade is higher and the patient cooperation is at its best.

By the investigations results, the oral health of PD patients can be considered generally worse than the comparable general population. This suggests that PD patients should follow a preferential routinely check dental care visit. Those procedures are fundamental for preventing the increasing of caries and periodontal diseases in those patients. 

Parkinson's disease is a progressive central nervous system disorder characterized by tremors, rigidity, and impaired motor function. Oral involvement is significant and affects the oral health status of the patient. According to this study, the frequencies of caries and periodontal disease are high in PD patients. Clinicians should routinely check those patients' oral health in order to maintain high quality of life of those patients. We suggest short and frequent dental visits for having high range attention of the PD patients. Moreover a strong dental hygiene education should be led on the PD patients in order to avoid the increase of invasive caries and to control active periodontal disease.
